# The American Public Health Association and Veterinary Medicine

**Published:** 1882-01

**Authors:** 


THE AMERICAN PUBLIC HEALTH ASSOCIATION AND VETERINARY MEDICINE.—The American Public Health Association is a national organization which, though young, already has gained a wide reputation through the public spirit and scientific attainments of its members. Its annual meetings are now watched with interest by the laity as well as the medical profession. It is a noteworthy fact, therefore, that at the annual meeting in November last, one out of the three days' session was devoted to comparative medicine. The list of papers read opened with one by Dr. Ezra M. Hunt, on "Contagious Diseases Among Domestic Animals." We publish it in another part of the JOURNAL, and gladly commend it to our readers as one of the most complete presentations of the claims of comparative medicine upon the State that has yet appeared.

The value of studies in comparative pathology, which has lately been so brilliantly demonstrated by Pasteur and others, is receiving every day a wider recognition. Articles upon this subject have lately appeared in the *Lancet*, the *N. Y. Medical Record*, the *Philadelphia Reporter*, the *College and Clinical Record*, and other journals. The *Southern Clinic* has established a veterinary department in its columns. A glance at the medical periodical literature of the different countries will show how frequently facts in comparative pathology are drawn upon or are contributed by students of the medical sciences.

